# Isolation and Characterization of a *Bacillus amyloliquefaciens* Bacteriophage JBA6 and Its Endolysin PlyJBA6

**DOI:** 10.4014/jmb.2502.02026

**Published:** 2025-04-01

**Authors:** Jena Kim, Minsuk Kong

**Affiliations:** Department of Food Science and Biotechnology, Research Institute of Food and Biotechnology, Seoul National University of Science and Technology, Seoul 01811, Republic of Korea

**Keywords:** *Bacillus amyloliquefaciens*, bacteriophage, endolysin, cell wall-binding domain

## Abstract

*Bacillus amyloliquefaciens* is a Gram-positive, aerobic, spore-forming bacteria usually found in soil. Despite its probiotic potential, *B. amyloliquefaciens* has been identified as a cause of food spoilage, including the development of off-odors, rope formation, and the production of viscous substances in a wide range of foods. To control *B. amyloliquefaciens*, we isolated three *B. amyloliquefaciens* bacteriophages TBA3, JBA3, JBA6, and characterized one representative JBA6 endolysin, PlyJBA6. Transmission electron microscopy and genomic analysis demonstrated that all three phages belong to the *Salasmaviridae* family, characterized by short, non-contractile tails with linear dsDNA genomes ranging from 18.7 to 19.1 kb. PlyJBA6 contains a glycoside hydrolase family 24 domain (PF00959) at the N-terminus and two LysM domains (PF04176) at the C-terminus. While JBA6 has a narrow host range, infecting only 7 out of 9 tested strains of *B. amyloliquefaciens*, PlyJBA6 exhibits extended lytic range beyond the *Bacillus* genus. Interestingly, PlyJBA6 lyses Gram-negative bacteria such as *Escherichia coli*, *Yersinia enterocolitica* and *Cronobacter sakazakii* without other additives to destabilize bacterial outer membrane. We assume that JBA6 might be a useful component for a phage cocktail to control *B. amyloliquefaciens* and that PlyJBA6 can provide insights into the development of novel biocontrol agents against various food-borne pathogens.

## Introduction

*Bacillus amyloliquefaciens* is a Gram-positive, aerobic, spore-forming bacterium widely utilized in agricultural and industrial applications due to its beneficial properties, including plant growth promotion, synthesis of bioactive compounds, and fermentation [1-3]. Although certain strains of *B. amyloliquefaciens* are used as probiotics and prebiotics in the food industry due to their antimicrobial activity [4, 5], some strains are associated with food spoilage, particularly in bakery products [6]. Ropy bread, a microbial spoilage phenomenon characterized by the development of an unpleasant fruity odor and subsequent enzymatic degradation of the crumb, leading to a soft and sticky texture, is commonly attributed to the growth of *B. amyloliquefaciens* [7]. Since bread has been a staple food for humans for thousands of years, spoilage remains a significant concern for both consumers and producers due to safety risks and economic losses [8]. Given that *B. amyloliquefaciens* is a spore-forming bacterium that is difficult to control, there is growing demand for the development of alternative methods to prevent food spoilage caused by *B. amyloliquefaciens*.

In response to the challenges posed by food spoilage bacteria, the use of biological control methods, such as bacteriophages and endolysins, has garnered increasing attention [9]. Bacteriophages are viruses that specifically infect bacterial cells, providing a highly targeted and efficient means of controlling bacterial populations. Unlike broad-spectrum antimicrobial agents, bacteriophages infect only specific bacterial species or strains, thereby minimizing disruption to beneficial microbiota [10]. Endolysins are phage-encoded enzymes that degrade the host cell wall, resulting in the release of phage progeny [11]. Endolysins derived from Gram-positive bacteria are typically composed of two distinct domains: an enzymatic activity domain (EAD) at the N-terminus and a cell wall binding domain (CBD) at the C-terminus [12]. The EAD is responsible for hydrolyzing the peptidoglycan layer, enabling bacterial cell lysis, while the CBD ensures binding specificity to the bacterial cell wall. Although both bacteriophages and endolysins have demonstrated significant potential as biocontrol agents, there are only a few studies reporting phages or endolysins targeting *B. amyloliquefaciens* [13, 14].

In this study, we isolated three closely-related bacteriophages TBA3, JBA3 and JBA6 and characterized one representative JBA6 endolysin, PlyJBA6. While these phages exhibited specific host ranges infecting certain *B. amyloliquefaciens* *and* *B. subtilis* strains, PlyJBA6 exhibited lytic activity against diverse Gram-positive bacteria, and notably, it also demonstrated antimicrobial effects against Gram-negative bacteria without the need for additives to destabilize bacterial outer membrane. This broad lytic activity may be attributed to cell wall-binding domain of PlyJBA6, which showed a wide binding spectrum across various bacterial genera. This study underscores the potential of PlyJBA6 as a biocontrol agent for managing food spoilage caused by *B. amyloliquefaciens* and possibly other foodborne pathogens, presenting a promising alternative to traditional chemical preservatives.

## Materials and Methods

### Bacterial Strains and Cultivation Media

The bacterial strains and culture conditions used in this study are listed in Table S1. *B. amyloliquefaciens* KACC 12067, 15877, and 19163 were used as the host and propagation strain of *B. amyloliquefaciens*-bacteriophages TBA3, JBA3, and JBA6, respectively. The agar media were prepared by supplementing the broth medium with 1.5% agar, and the soft agar medium was formulated using distilled water (DW) containing 0.7% agar. All the media used in this study were purchased from BD Difco (USA).

### Bacteriophage Isolation and Purification

*B. amyloliquefaciens*-infecting phages were isolated from sludge samples (Jungnang Water Reclamation Center, Republic of Korea) or horse manure samples (Seoul Horse Riding Club, Republic of Korea). The method described in [15] was followed with slight modifications. 10 ml of each sample was mixed with an equal volume of 2X BHI broth containing 200 μl of an overnight culture of *B. amyloliquefaciens*, supplemented with 5 mM MgCl_2_ and CaCl_2_. After initial isolation, phage propagation and purification were conducted following the methods described in the previous study [16].

### Host Range Analysis

Host range analysis was performed as previously described [17]. Briefly, the bacteriophage stocks (approximately 10^10^-10^11^ PFU/ml) were 10-fold serially diluted in SM buffer. 10 μl of aliquots were spotted onto the 0.7% molten top agar plates containing each of bacterial cells and the plates were incubated overnight. Phage titers were determined as plaque-forming units (PFU/ml).

### Transmission Electron Microscopy (TEM)

Morphological analysis of purified bacteriophages was performed using TEM [18]. Drops of purified bacteriophages (10^10^ PFU/ml) in SM buffer were applied to formvar/carbon-coated copper grids (200 mesh; EMS, USA) after a glow-discharge preparation conducted at 15 mA and 0.26 mBar for 30 sec using a glow discharge cleaning system (PELCO easiGlow, Ted Pella Inc., USA). The grids containing the phage particles were negatively stained with 2% (w/v) uranyl acetate (pH 4.0) and visualized using Energy-Filtering TEM (EF-TEM) at 80 kV (NICEM, Republic of Korea). Bacteriophages were classified into their relative family according to the International Committee on Taxonomy of Viruses (ICTV) guidelines based on the morphology of phages [19].

### Genome Sequencing and *in silico* Analysis

Phage DNA was extracted using the phage DNA isolation kit as the manufacturer’s instructions (Norgen Biotek, Co., Canada). The purified phage genomic DNA was sequenced with the Illumina Novaseq 6000 platform (LabGenomics, Republic of Korea) and assembled with metaSPAdes v.3.14.1 (Sanigen, Republic of Korea). First-pass genome annotation was performed using RAST (Rapid Annotation using Subsystem Technology) pipeline. Conserved domains were identified using InterProScan [20] and the NCBI's Conserved Domain Database (CDD)[21] analysis. Basic Local Alignment Search Tool for Proteins (BLASTP) [22] analysis was performed to assign functional annotations to the predicted ORFs. The presence of genes encoding tRNAs was screened using the tRNAscan-SE database [23]. Based on the information, each ORF’s name was manually annotated, and the complete linear genome and predicted ORFs were visualized into a circular map with CGView server [24]. Comparative whole genome sequence analysis of phages (TBA3, JBA3, JBA6, phi29 [25], vB_BveP-Goe6 [26], and Gxv1 [27]) was done using Easyfig software ver.2.2.5. [28]. The tertiary structure of PlyJBA6 was predicted using the AlphaFold2 [29] with predicted local distance difference test (pLDDT) scores, and viewed using iCn3D server [30].

### Phylogenetic Tree

To study the relationship between *Bacillus* phages, a phylogenetic tree was constructed. The sequences of some “core” genes encoding major capsid protein (MCP) and the large terminase protein subunit (TerL) were used to examine the phylogenetic location of phage JBA6. Over 30 of the genomes of the *Bacillus* phages were downloaded from the NCBI database. Amino acid sequences of MCP or TerL among these phages were aligned using ClustalW [31], and the phylogenetic tree was constructed using the neighbor-joining analysis and finally visualized using MEGA11 [32].

### Cloning, Expression, and Purification of PlyJBA6

The endolysin gene (PlyJBA6) and its cell wall binding domain (PlyJBA6_CBD) were amplified from the genomic DNA of the phage JBA6 by polymerase chain reaction (PCR) with FastPfu DNA polymerase (TransStart, China). The primers used in PCR reaction are listed in Table S2. The gene encoding PlyJBA6, digested with NcoI and SalI restriction enzymes was inserted into the pET28a vector (Novagen, USA). The gene encoding PlyJBA6_CBD was amplified by PCR, digested with BamHI and HindIII restriction enzymes, and cloned in the EGFP::pET28a. The recombinant plasmid was transformed into competent *E. coli* BL21 (DE3) for protein expression. The protein expression and purification procedures were conducted as previously described [18]. The purified protein PlyJBA6 was stored in lysis buffer at -80°C while purified PlyJBA6_CBD was stored in a storage buffer at -20°C.

### Turbidity Reduction Assay and Lytic Range of PlyJBA6

The lytic activity of the recombinant endolysin PlyJBA6 against bacterial cells was assayed by monitoring the decrease in OD_600_, and the turbidity reduction assay was performed as previously described [18]. A decrease in OD_600_ was monitored using a multimode microplate reader (SpectraMax i3x, Molecular Device) for 60 min with 1 min intervals at 25°C. To determine the lytic range, 0.4 μM of PlyJBA6 were added to bacterial cells in reaction buffer (20mM Tris-Cl, pH 8.0). The lytic range of PlyJBA6 was represented by the relative lytic activity (%) using Eq. (1).

$$\text{Relative lytic activity (\%)}=\frac{100\%\times \left({\text{OD}}_{\text{600}}{\text{ of control-OD}}_{600}\text{ of the endolysin treatment}\right)}{{\text{the initial OD}}_{600}\text{ of the control}}$$ (1)

The effects of pH and NaCl on the enzymatic activity of PlyJBA6 were assessed, as previously described [18]. To determine the thermal stability, PlyJBA6 was preincubated at various temperatures for 20 min and its residual activity was measured by turbidity reduction assay.

### Cell-Binding Assay of EGFP:PlyJBA6_CBD

The cell wall-binding activity was observed with fluorescence microscopy as previously described [33]. Drops of sample (3 μl) were placed on the slide glass. A piece of agarose pad was put on a sample for cell immobilization. For fluorescence microscopy, images were captured using an inverted microscope (ECLIPSE Ti2, Nikon).

### Statistical Analysis

All experiments were done in triplicate. GraphPad Prism 5 was used for all statistical analysis. Data are means ± standard deviation (SD) from independent experiments.

### GenBank Accession Numbers

The nucleotide sequences of bacteriophage TBA3, JBA3, and JBA6 were deposited to GenBank under the accession number OM249958, OP204508, and OK625815, respectively.

## Results and Discussion

### Isolation of Three *B. amyloliquefaciens* Phages

**Fig. 1:**
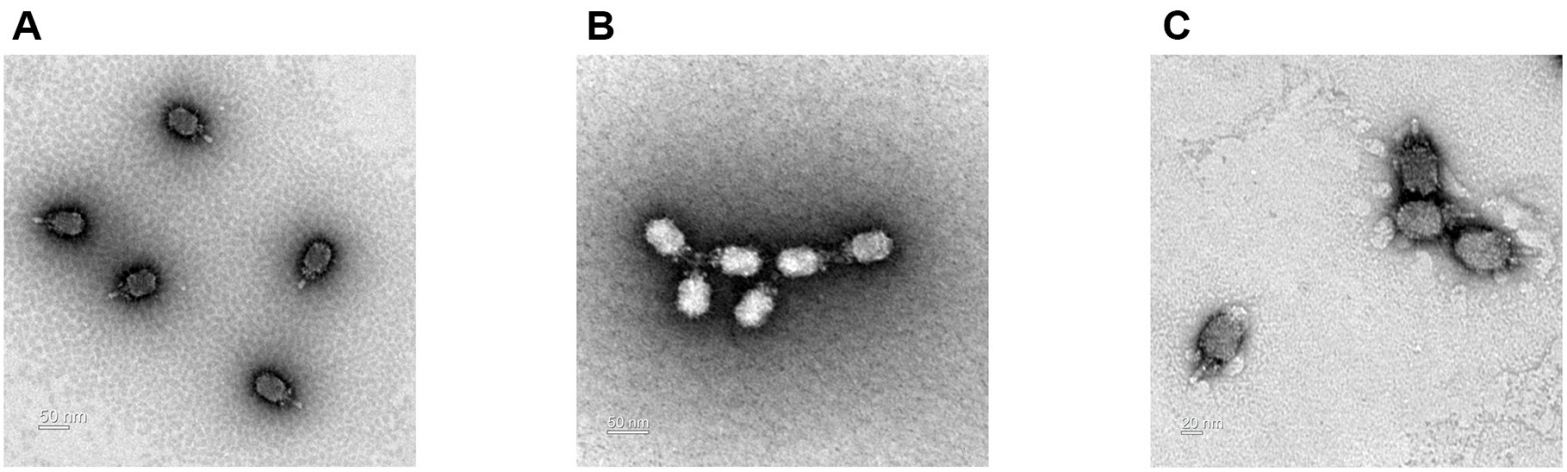
TEM analysis of three *B. amyloliquefaciens* phages. TBA3 (**A**), JBA3 (**B**), and JBA6 (**C**) belong to the family *Salasmaviridae*.

*B. amyloliquefaciens* phage JBA3 and JBA6 were isolated from a sewage sample, and phage TBA3 was isolated from horse manure (Fig. S1). TEM analysis showed that the three phages have similar morphology, having an icosahedral head with a short and non-contractile tail, characteristic of the *Caudoviricetes* class (Fig. 1). To determine host ranges of these phages, 16 species of Gram-positive bacteria and 9 species of Gram-negative bacteria, including *E. coli* and *Salmonella*, were used. The assay revealed that phages TBA3, JBA3, and JBA6 infect most strains of *B. amyloliquefaciens*, but it was unable to infect other species including *B. licheniformis*, *B. pumilus*, *B. megaterium*, *B. thuringiensis*, *B. mycoides*, and *B. cereus*, indicating that these phages showed high host specificity (Table 1).

**Fig. 2:**
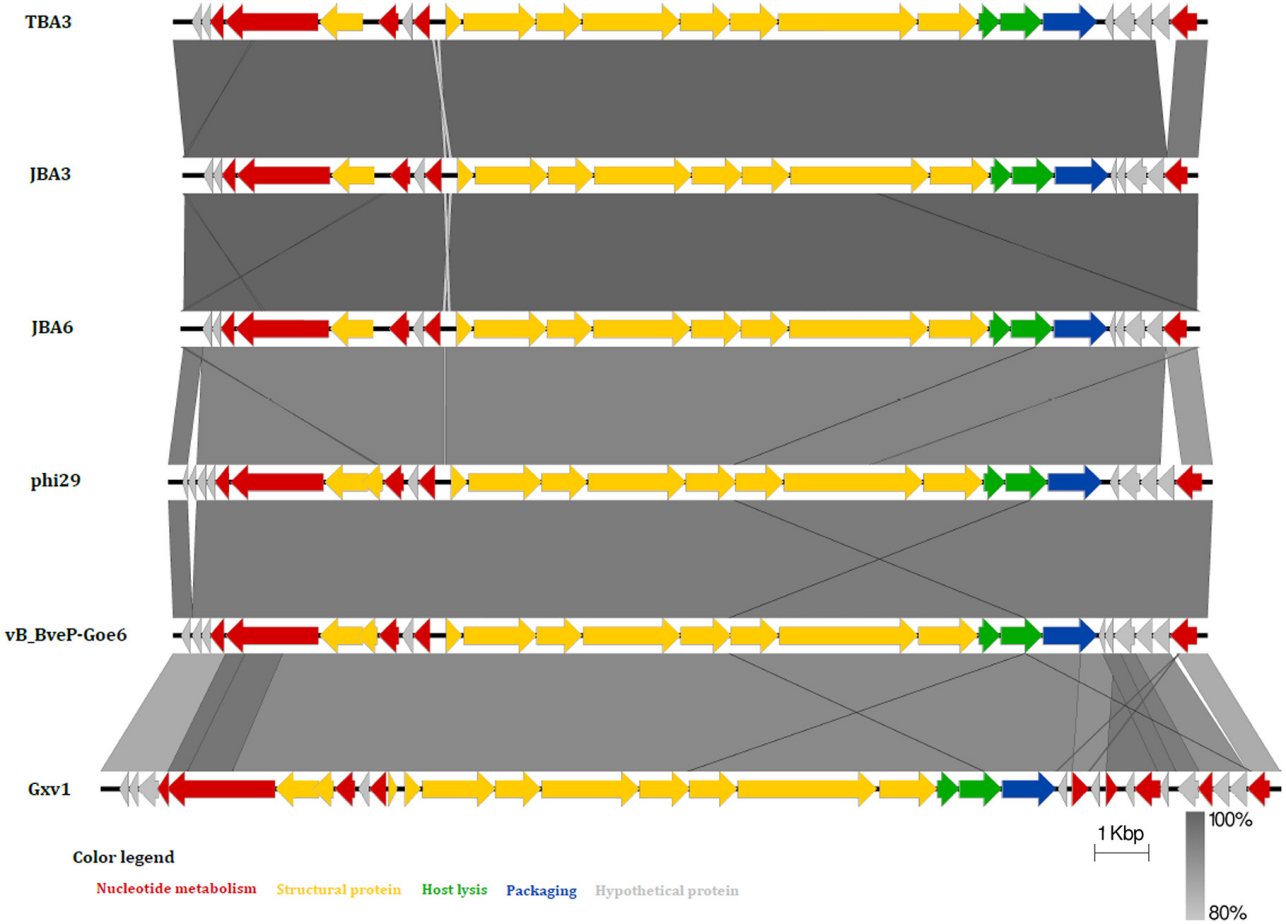
Comparative genomic analysis of among *Bacillus* phages TBA3, JBA3, JBA6, phi29, vB_BveP-Goe6, and Gxv1. Each color of ORFs indicates its function.

Genomic analysis revealed that all three phages have linear double-stranded DNA (dsDNA), varying in size from 18,752 to 19,105 bp containing 24 possible open reading frames (ORFs) (Fig. S2). The ORFs of phages TBA3, JBA3, and JBA6 were functionally categorized into five groups: nucleotide metabolism, structure, host lysis, packaging, and hypothetical proteins. The absence of lysogeny-related genes coding for integrase, recombinase, repressors, or excisionase, which are key markers of temperate viruses suggests that TBA3, JBA3, and JBA6 are lytic phages. Comparative genomic analysis revealed that these phages are closely related (99.9% identity) and share 93.7% nucleotide identity with *B. subtilis* phage phi29, 92.5% identity with *B. velezensis* phage vB_BveP-Goe6, and 94.5% identity with *B. velezensis* phage Gxv1 (Fig. 2). Among these six phages, only JBA3 and JBA6 lack the hypothetical gene (ORF2 of TBA3). Considering that both JBA3 and JBA6 share the same host range but differ from TBA3 in their host range (Table 1), this gene may be important for determining phage host specificity. Given the high similarity among the TBA3, JBA3, and JBA6 phages, JBA6 was selected as the representative for further phylogenetic analysis. Both phylogenetic trees, based on the terminase large subunit (TerL) and the major capsid protein (MCP), indicated that JBA6 clusters tightly with phages infecting *B. subtilis* and *B. velezensis* (Fig. S3). These findings indicated that JBA6 belongs to the *Salasmaviridae* family, supported by similar genome sizes, conserved genes, and phage morphologies observed via TEM.

### Characterization of PlyJBA6

**Fig. 3:**
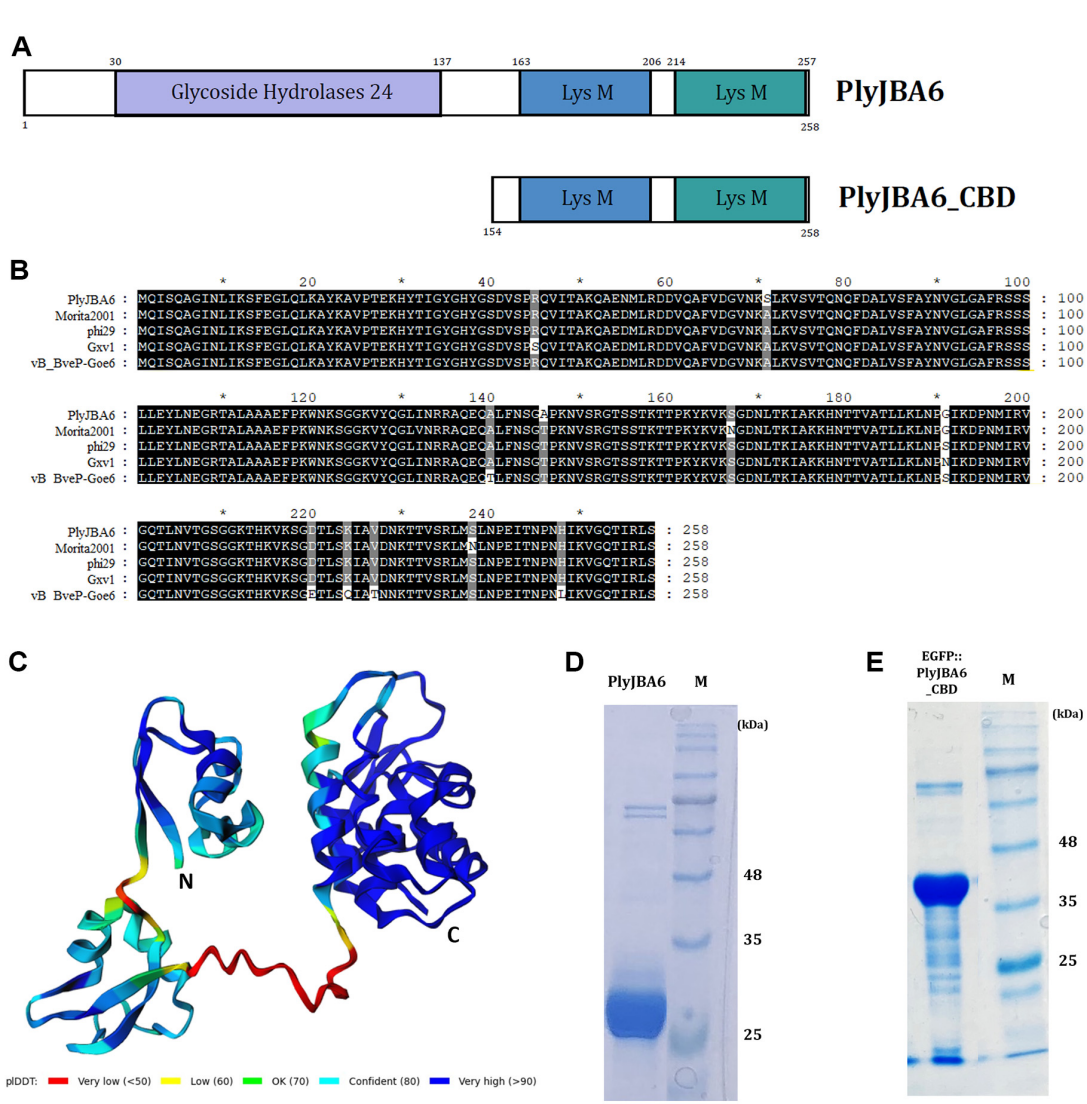
Modular structure of PlyJBA6. (**A**) Schematic representation of PlyJBA6 and PlyJBA6_CBD. Each number corresponds to the positions of amino acid residues. (**B**) Amino acid sequence alignment among endolysins of JBA6-like phages. Conserved and identical residues are shaded in gray (>70% conserved) and black, respectively. (**C**) Predicted threedimensional model of PlyJBA6 using AlphaFold2. pLDDT is coloured from blue (high confidence) to red or orange (low confidence). (**D, E**) SDS-PAGE analysis of purified PlyJBA6 (29.5 kDa) and EGFP::PlyJBA6_CBD (40.5 kDa). Lane M, protein weight marker.

The putative endolysin gene (ORF18) identified from the JBA6 genome was designated as PlyJBA6. As shown in Fig. 3A, CDD and BLASTP analyses showed that PlyJBA6 consists of an N-terminal family 24-glycoside hydrolase catalytic domain (PF00959) and two C-terminal LysM domains (PF04176). Amino acid sequence alignment demonstrated that PlyJBA6 is highly homologous to endolysins of *Bacillus* phages Morita2001, phi29, Gxv1, and vB_BveP-Goe6, (Fig. 3B). The three-dimensional model of PlyJBA6 using AlphaFold2 revealed high-confidence structural predictions (>90 pLDDT) for both the N-terminal glycoside hydrolase domain and the C-terminal LysM domains (Fig. 3C). In contrast, the central linker region exhibited lower confidence scores (<70 pLDDT), indicating its structural flexibility, which may enhance enzymatic efficiency and substrate recognition of PlyJBA6. We cloned the PlyJBA6 gene and expressed in *E. coli* with a C-terminal His-tag. Sodium dodecyl sulfate-polyacrylamide gel electrophoresis (SDS-PAGE) confirmed a single band of the purified PlyJBA6 protein (29.5 kDa) (Fig. 3D).

**Fig. 4:**
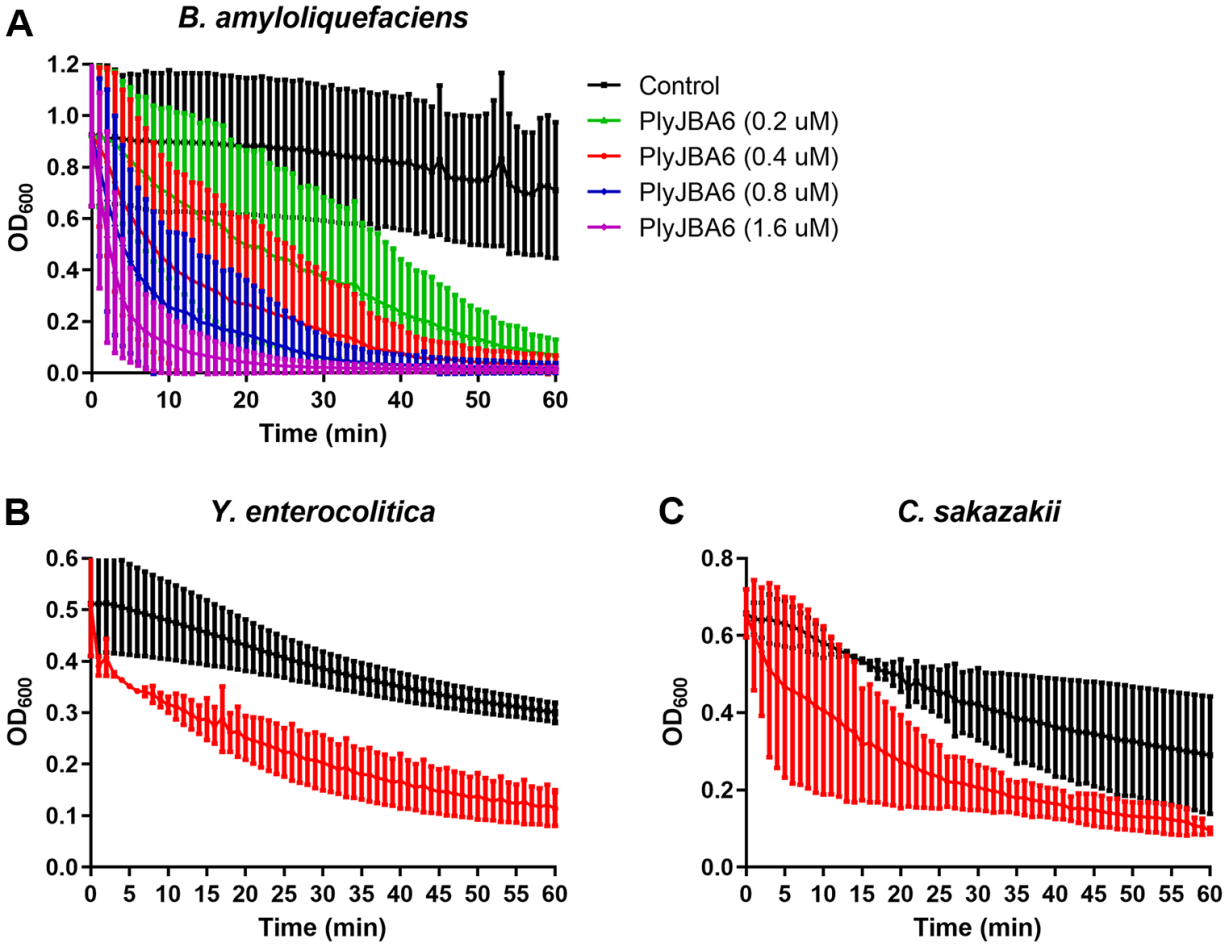
Lytic activity of PlyJBA6. (**A**) Different concentrations of PlyJBA6 were added to exponentially grown *B. amyloliquefaciens* KACC 15877 cells in reaction buffer and a decrease in OD_600_ was measured for 60 min in 1 min intervals. Turbidity reduction of *Y. enterocolitica* ATCC 55075 (**B**) and *C. sakazakii* ATCC 29544 (**C**) cells in the presence of 0.4 μM PlyJBA6.

Turbidity reduction assay demonstrated that PlyJBA6 showed dose-dependent lysis activity against *B. amyloliquefaciens* KACC 15877 cells (Fig. 4A). While phage JBA6 specifically infects *B. amyloliquefaciens* strains, PlyJBA6 exhibited a much broader lytic spectrum including pathogenic Gram-positive bacteria, such as *Bacillus cereus*, *Listeria monocytogenes* (Table 1). Remarkably, PlyJBA6 also showed lytic activity against Gram-negative bacteria, including *Yersinia entercolitica*, *Cronobacter sakazakii* (Fig. 4B and 4C), without the use of outer membrane permeabilizer treatment such as ethylenediaminetetraacetic acid (EDTA). Gram-negative bacteria are typically resistant to lytic actions of endolysins due to their protective outer membrane. To overcome this limitation, many researchers have attempted to fuse endolysins with cationic antimicrobial peptides or combine endolysins with organic acids/essential oils to enhance endolysin permeability through the outer membrane [9]. Since PlyJBA6 is a highly basic protein (pI = 10.05), the net positive charge could interact with the negatively charged lipopolysaccharide (LPS) layer of the Gram-negative outer membrane. It is also possible that the C-terminal cationic region of PlyJBA6 fold into amphipathic structures, destabilizing LPS or form pores in the membrane bilayer [34]. Notably, *in silico* analysis indicated that the C-terminal LysM domains of PlyJBA6, rich in positively charged amino acids surrounded by hydrophobic amino acids, could form amphipathic α-helixes, leading to the destabilization of outer membrane and enhanced access of endolysins to peptidoglycan [35]. Although further studies are required to elucidate the molecular mechanism underlying its intrinsic lytic activity of PlyJBA6, this result could aid the development of effective antimicrobial agents against Gram-negative pathogens.

**Fig. 5:**
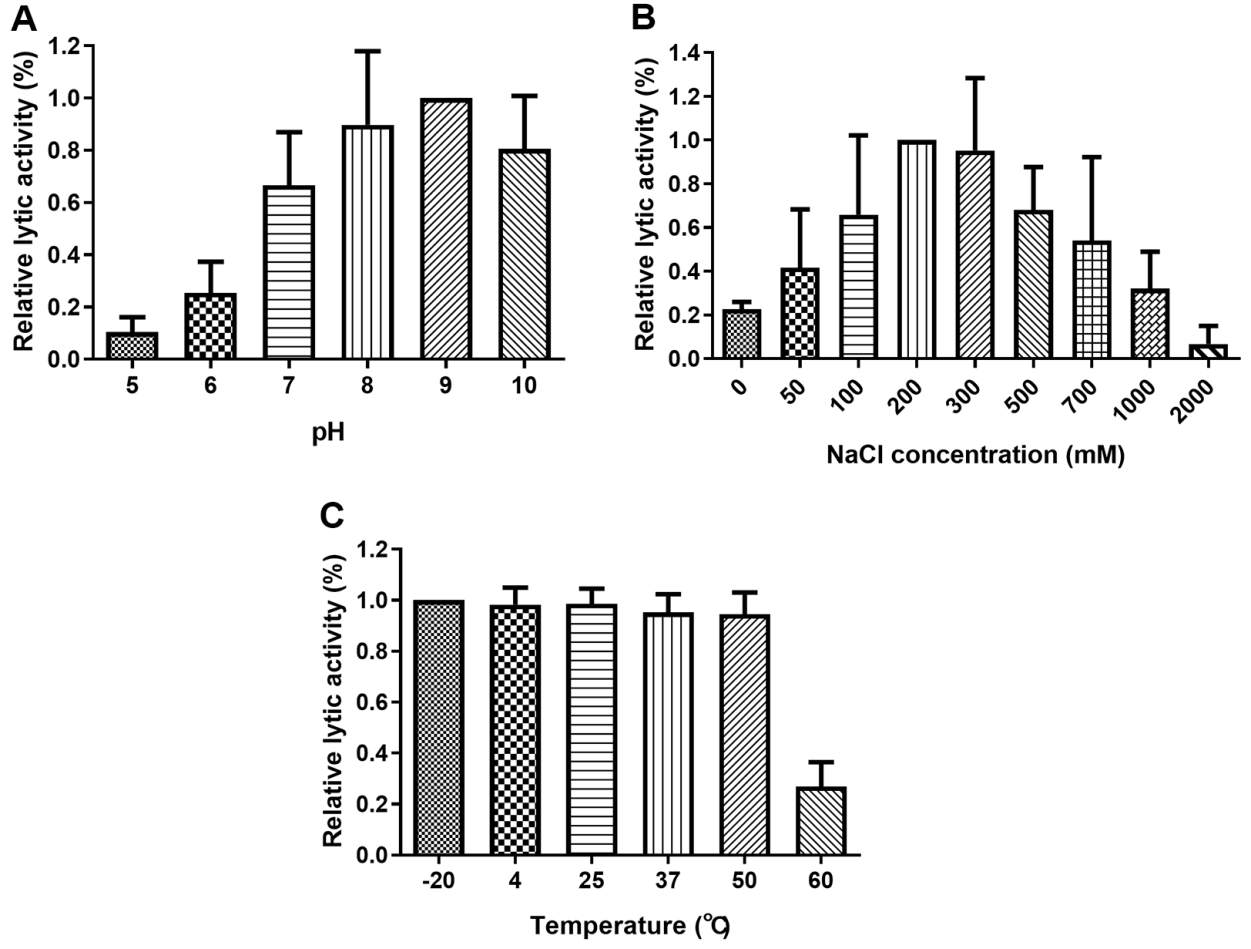
Biochemical properties of PlyJBA6. The effects of pH (**A**), NaCl (**B**) on the lytic activity of PlyJBA6 against *B. amyloliquefaciens* cells. The relative lytic activity was normalized to the activity displayed by the maximal activity group. (**C**) Thermal stability of PlyJBA6. The residual activity of PlyJBA6 after thermal treatment (20 min) was measured using turbidity reduction assay.

To determine the effects of NaCl, pH, and temperature on the lytic activity of PlyJBA6, its relative activity was evaluated under various environmental conditions. PlyJBA6 maintained its lytic activity in alkaline conditions, while only marginal activity was detectable under pH 6.0 (Fig. 5A). This is interesting because Morita2001 endolysin (97% amino acid sequence identity to PlyJBA6) showed maximum activity at pH 5 and rapidly lost activity above pH 6 [13]. Further studies will be necessary to identify the residues that contribute to the different pH profiles between the two closely-related endolysins. PlyJBA6 also exhibited its activity across NaCl concentrations up to 1000 mM (Fig. 5B). As shown in Fig. 5C, PlyJBA6 maintains full activity up to 50°C, with a sharp decline observed at 60°C. These biochemical properties of PlyJBA6 may provide a foundation for its use in diverse environmental conditions.

### CBD from Endolysin PlyJBA6

**Fig. 6:**
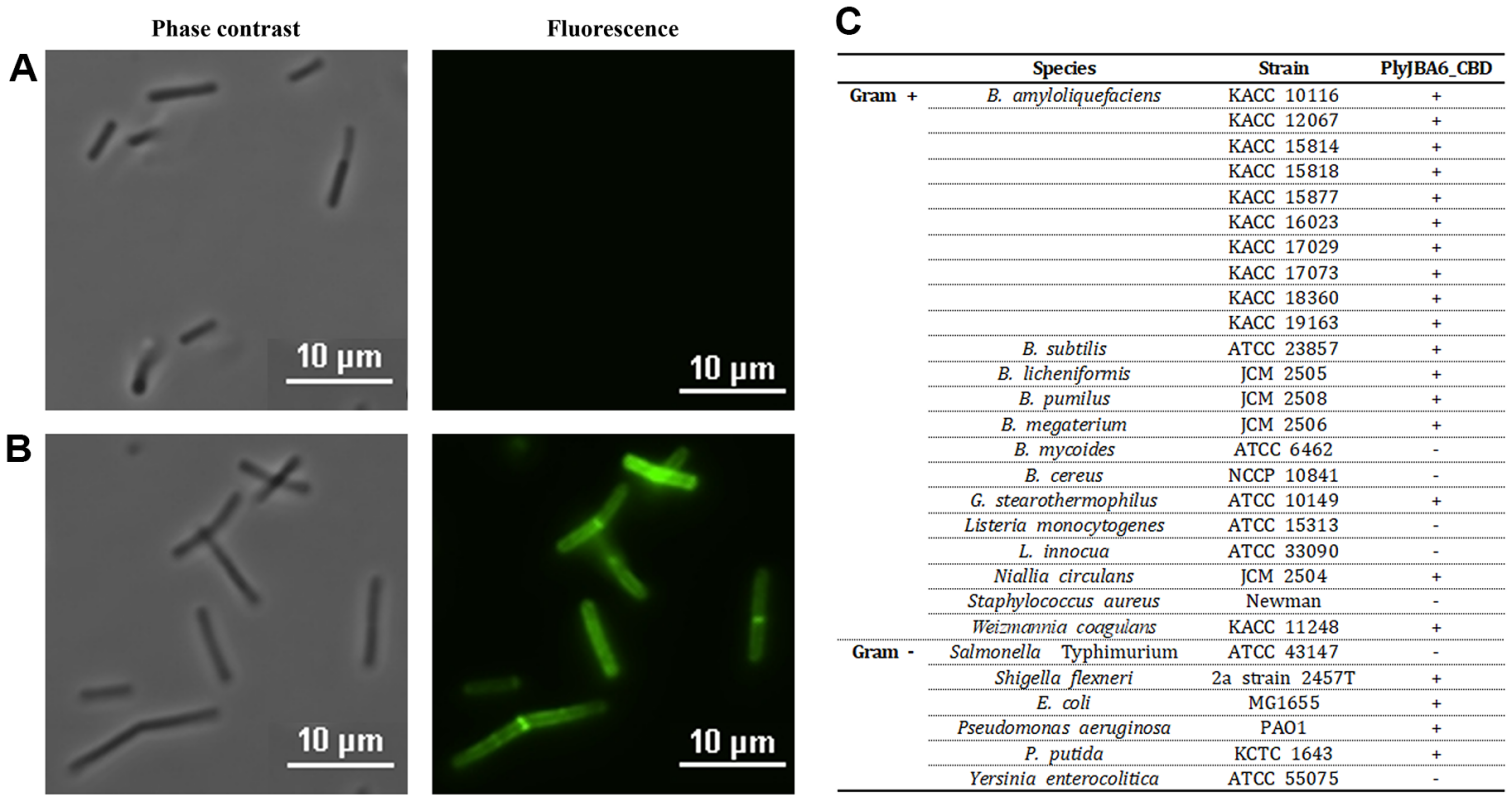
Cell-wall binding activity of PlyJBA6_CBD. Phase contrast and fluorescence microscopy images of *B. amyloliquefaciens* cells treated with EGFP only (**A**) and EGFP:: PlyJBA6_CBD (**B**) in PBS buffer. (**C**) Binding spectrum of PlyJBA6_CBD.

Although the Morita 2001 endolysin demonstrated a broad antibacterial spectrum, including several Gram-negative bacteria, and its C-terminal region has been proposed to be responsible for enhancing outer membrane permeability, direct interactions between the endolysin and a wide range of bacterial cells were not observed [13, 14]. To confirm the bacterial cell-wall binding activity of the C-terminal LysM domains of PlyJBA6, we fused the gene encoding 154-258 amino acid residues of PlyJBA6 (hereafter called PlyJBA6_CBD) with enhanced green fluorescence protein (EGFP) and expressed in *E. coli*. The SDS-PAGE result showed a single band of purified EGFP:PlyJBA6_CBD (40.5 kDa) (Fig. 3E). The fluorescence microscopy analysis revealed that EGFP-only proteins showed no binding activity, but EGFP::PlyJBA6_CBD showed strong binding toward *B. amyloliquefaciens* cells (Fig. 6A and 6B). EGFP::PlyJBA6_CBD also binds to various bacteria, including *Bacillus megaterium*, *Geobacillus stearothermophilus*, *Weizmannia coagulans*, *Pseudomonas aeruginosa*, and *Escherichia coli* (Figs. 6C and S4), suggesting that PlyJBA6_CBD targets broadly conserved cell wall structure of these bacteria. Interestingly, PlyJBA6_CBD did not bind to Listeria cells that are highly susceptible to PlyJBA6. This result indicated that the lytic activity of PlyJBA6 does not necessarily depend on the CBD binding to cognate target cells.

LysM domains, commonly found in phage endolysins and bacterial autolysins, are known to bind to N-acetylglucosamine moiety of peptidoglycan [36]. Several LysM domain-containing endolysins have been reported to exhibit a broad lytic and binding spectrum against both Gram-positive and Gram-negative bacteria. The LysM domain of *Lactobacillus fermentum* bacteriophage endolysin Lyb5 was able to bind to a broad range of lactic acid bacteria, including *Lactococcus*, *Lactobacillus*, and *Streptococcus* [37]. The LysM domains of *Geobacillus stearothermophilus* bacteriophage GR1 endolysin could bind to *Geobacillus*, *Bacillus*, *Weizmannia*, and *Clostridium* [18]. In the case of PlyJBA6, two C-terminal LysM domains function as the CBD and have broad binding spectrum across various bacterial genera, including several Gram-negative bacteria. Although it remains to be investigated whether PlyJBA6_CBD destabilizes the outer membrane and target the endolysins to the peptidoglycan of Gram-negative bacteria, this result suggests that PlyJBA6_CBD has the potential to be developed as a novel antibacterial agent against Gram-negative bacteria.

In conclusion, we isolated three JBA6-like virulent bacteriophages that infect food spoilage bacterium, *B. amyloliquefaciens*. An endolysin gene, plyJBA6 was identified in the genome of JBA6 and further characterized. PlyJBA6 demonstrated a broad antimicrobial spectrum and can even lyse Gram-negative bacteria without any outer membrane permeabilizers. This broad lytic activity could be partially attributed to the presence of C-terminal LysM domains which function as a PlyJBA6_CBD showing wide binding spectrum. These results suggest that PlyJBA6 and PlyJBA6_CBD could be used as novel sources for the development of efficient antimicrobials against various pathogens including Gram-negative bacteria.

## Supplemental Materials

Supplementary data for this paper are available on-line only at http://jmb.or.kr.



## Figures and Tables

**Table 1:** The antimicrobial spectrum of phage TBA3, JBA3, JBA6, its endolysin PlyJBA6.

	Species	Strain^a^	TBA3	JBA3	JBA6	PlyJBA6^b^
Gram +	*B. amyloliquefaciens*	KACC 10116	-	-	-	+++
		KACC 12067	+	+	+	+++
		KACC 15814	+	-	-	+++
		KACC 15818	+	+	+	+++
		KACC 15877	-	+	+	+++
		KACC 16023	+	+	+	+++
		KACC 17029	+	+	+	+++
		KACC 17073	+	+	+	+++
		KACC 18360	-	-	-	++
		KACC 19163	+	+	+	+++
	*B. subtilis*	ATCC 23857	-	+	+	+++
	*B. licheniformis*	JCM 2505	-	-	-	++
	*B. pumilus*	JCM 2508	-	-	-	++
	*B. megaterium*	JCM 2506	-	-	-	++
	*B. thuringiensis*	ATCC 10792	-	-	-	-
	*B. mycoides*	ATCC 6462	-	-	-	-
	*B. cereus*	NCCP 10634	-	-	-	++
		NCCP 10841	-	-	-	-
	*Levilactobacillus brevis*	KACC 10553	-	-	-	-
	*G. stearothermophilus*	ATCC 10149	-	-	-	+
	*Clostridium perfringens*	NCCP 15911	-	-	-	-
	*Listeria monocytogenes*	ATCC 15313	-	-	-	++
	*L. innocua*	ATCC 33090	-	-	-	++
	*Niallia circulans*	JCM 2504	-	-	-	-
	*Staphylococcus aureus*	Newman	-	-	-	-
	*Weizmannia coagulans*	KACC 11248	-	-	-	++
Gram -	*Salmonella* Enteritidis	ATCC 13076	-	-	-	-
	*S*. Typhimurium	ATCC 43147	-	-	-	+
	*Shigella flexneri*	2a strain 2457T	-	-	-	-
	*E. coli* O157:H7	ATCC 35150	-	-	-	-
	*E. coli*	MG1655	-	-	-	++
	*Pseudomonas aeruginosa*	PAO1	-	-	-	+
		ATCC 27853	-	-	-	-
	*P. putida*	KCTC 1643	-	-	-	+
	*Yersinia enterocolitica*	ATCC 55075	-	-	-	+
	*Cronobacter sakazakii*	ATCC 29544	-	-	-	++

^a^ATCC, American Type Culture Collection; KACC, Korean Agricultural Culture Collection; KCTC, Korean Collection for Type Culture; JCM, Journal of Clinical Medicine; NCCP, National Culture Collection for Pathogens

^b^The percentage of lytic activity was obtained by the turbidity reduction assay for 60 min; -: 0-10%; +: 11-40%; ++: 41-70%; +++: 71-100%.

## References

[1] Yang P, Yuan P, Liu W, Zhao Z, Bernier MC, Zhang C (2024). Plant growth promotion and plant disease suppression induced by *Bacillus amyloliquefaciens* strain GD4a. Plants.

[2] Luo L, Zhao C, Wang E, Raza A, Yin C (2022). *Bacillus amyloliquefaciens* as an excellent agent for biofertilizer and biocontrol in agriculture: an overview for its mechanisms. Microbiol. Res..

[3] WoldemariamYohannes K, Wan Z, Yu Q, Li H, Wei X, Liu Y (2020). Prebiotic, probiotic, antimicrobial, and functional food applications of *Bacillus amyloliquefaciens*. J. Agric. Food Chem..

[4] Du H, Yao W, Kulyar MF-e-A, Ding Y, Zhu H, Pan H (2022). Effects of *Bacillus amyloliquefaciens* TL106 isolated from Tibetan pigs on probiotic potential and intestinal microbes in weaned piglets. Microbiol. Spectr..

[5] Zeng Z, He X, Li F, Zhang Y, Huang Z, Wang Y (2021). Probiotic properties of *Bacillus proteolyticus* isolated from Tibetan yaks, China. Front. Microbiol..

[6] Pacher N, Burtscher J, Johler S, Etter D, Bender D, Fieseler L (2022). Ropiness in bread-a re-emerging spoilage phenomenon. Foods.

[7] Valerio F, Di Biase M, Huchet V, Desriac N, Lonigro S, Lavermicocca P (2015). Comparison of three *Bacillus amyloliquefaciens* strains growth behaviour and evaluation of the spoilage risk during bread shelf-life. Food Microbiol..

[8] Rahman M, Islam R, Hasan S, Zzaman W, Rana MR, Ahmed S (2022). A comprehensive review on bio-preservation of bread: an approach to adopt wholesome strategies. Foods.

[9] Choi D, Ryu S, Kong M (2025). Phage‐derived proteins: advancing food safety through biocontrol and detection of foodborne pathogens. Compr. Rev. Food Sci. Food Saf..

[10] Lee C, Kim H, Ryu S (2023). Bacteriophage and endolysin engineering for biocontrol of food pathogens/pathogens in the food: recent advances and future trends. Crit. Rev. Food Sci. Nutr..

[11] Schmelcher M, Loessner MJ (2016). Bacteriophage endolysins: applications for food safety. Curr. Opin. Biotechnol..

[12] Oliveira H, Melo LD, Santos SB, Nóbrega FL, Ferreira EC, Cerca N (2013). Molecular aspects and comparative genomics of bacteriophage endolysins. J. Virol..

[13] Morita M, Tanji Y, Mizoguchi K, Soejima A, Orito Y, Unno H (2001). Antibacterial activity of *Bacillus amyloliquefaciens* phage endolysin without holin conjugation. J. Biosci. Bioeng..

[14] Orito Y, Morita M, Hori K, Unno H, Tanji Y (2004). *Bacillus amyloliquefaciens* phage endolysin can enhance permeability of *Pseudomonas aeruginosa* outer membrane and induce cell lysis. Appl. Microbiol. Biotechnol..

[15] Kong M, Ryu S (2015). Bacteriophage PBC1 and its endolysin as an antimicrobial agent against *Bacillus cereus*. Appl. Environ. Microbiol..

[16] Kong M, Na H, Ha N-C, Ryu S (2019). LysPBC2, a novel endolysin harboring a *Bacillus cereus* spore binding domain. Appl. Environ. Microbiol..

[17] Na H, Kong M, Ryu S (2016). Characterization of LysPBC4, a novel *Bacillus cereus*-specific endolysin of bacteriophage PBC4. FEMS Microbiol. Lett..

[18] Choi D, Kong M (2023). LysGR1, a novel thermostable endolysin from *Geobacillus stearothermophilus* bacteriophage GR1. Front. Microbiol..

[19] Walker PJ, Siddell SG, Lefkowitz EJ, Mushegian AR, Adriaenssens EM, Alfenas-Zerbini P (2022). Recent changes to virus taxonomy ratified by the international committee on taxonomy of viruses (2022). Arch. Virol..

[20] Jones P, Binns D, Chang H-Y, Fraser M, Li W, McAnulla C (2014). InterProScan 5: genome-scale protein function classification. Bioinformatics.

[21] Lu S, Wang J, Chitsaz F, Derbyshire MK, Geer RC, Gonzales NR (2020). CDD/SPARCLE: the conserved domain database in 2020. Nucleic Acids Res..

[22] Camacho C, Coulouris G, Avagyan V, Ma N, Papadopoulos J, Bealer K (2009). BLAST+: architecture and applications. BMC Bioinformatics.

[23] Chan PP, Lowe TM (2019). tRNAscan-SE: searching for tRNA genes in genomic sequences. Methods Mol. Biol..

[24] Grant JR, Stothard P (2008). The CGView Server: a comparative genomics tool for circular genomes. Nucleic Acids Res..

[25] Meijer WJ, Horcajadas JA, Salas M (2001). Phi29 family of phages. Microbiol. Mol. Biol. Rev..

[26] Schilling T, Hoppert M, Daniel R, Hertel R (2018). Complete genome sequence of vB_BveP-Goe6, a virus infecting *Bacillus velezensis* FZB42. Genome Announc..

[27] Guo X, Zhang T, Jin M, Zeng R (2021). Characterization of *Bacillus* phage Gxv1, a novel lytic *Salasvirus* phage isolated from deep-sea seamount sediments. Mar. Life Sci. Technol..

[28] Sullivan MJ, Petty NK, Beatson SA (2011). Easyfig: a genome comparison visualizer. Bioinformatics.

[29] Jumper J, Evans R, Pritzel A, Green T, Figurnov M, Ronneberger O (2021). Highly accurate protein structure prediction with AlphaFold. Nature..

[30] Wang J, Youkharibache P, Zhang D, Lanczycki CJ, Geer RC, Madej T (2020). iCn3D, a web-based 3D viewer for sharing 1D/2D/3D representations of biomolecular structures. Bioinformatics.

[31] Sievers F, Higgins DG (2021). The clustal omega multiple alignment package. Methods Mol. Biol..

[32] Tamura K, Stecher G, Kumar S (2021). MEGA11: molecular evolutionary genetics analysis version 11. Mol. Biol. Evol..

[33] Yu B, Kong M (2025). Characterization of an endolysin from B. cereus-infecting bacteriophage B13S and its application as antimicrobial and detection agents. LWT.

[34] Brogden KA (2005). Antimicrobial peptides: pore formers or metabolic inhibitors in bacteria?. Nat. Rev. Microbiol..

[35] Sisson HM, Jackson SA, Fagerlund RD, Warring SL, Fineran PC (2024). Gram-negative endolysins: overcoming the outer membrane obstacle. Curr. Opin. Microbiol..

[36] Visweswaran GRR, Leenhouts K, van Roosmalen M, Kok J, Buist G (2014). Exploiting the peptidoglycan-binding motif, LysM, for medical and industrial applications. Appl. Microbiol. Biotechnol..

[37] Hu S, Kong J, Kong W, Guo T, Ji M (2010). Characterization of a novel LysM domain from *Lactobacillus fermentum* bacteriophage endolysin and its use as an anchor to display heterologous proteins on the surfaces of lactic acid bacteria. Appl. Environ. Microbiol..

