# Supplementary material for: Isolation and Characterization of a Bacillus amyloliquefaciens Bacteriophage JBA6 and Its Endolysin PlyJBA6
Source: J Microbiol Biotechnol. 2025 Apr 1;35:e2502026. doi: 10.4014/jmb.2502.02026 (PMC11985411; doi:10.4014/jmb.2502.02026)
Supplement: Supplementary file 1 [file jmb-35-e2502026-supple.pdf]

J. Microbiol. Biotechnol. 2025. 35: e2502026

https://doi.org/10.4014/jmb.2502.02026

Supplementary Tables and Figures

Table S1. Growth condition and cultivation media of bacteria used in this study.

**Species**	**Growth condition**	**Cultivation Media**
Bacillus amyloliquefaciens	30℃ / 250 rpm / aerobic	Brain Heart Infusion (BHI)
B. subtilis, B. licheniformis, B. pumilus, B. circulans, Listeria monocytogenes, L. innocua	37℃ / 250 rpm / aerobic	Brain Heart Infusion (BHI)
B. cereus, B. megaterium, B. thuringiensis, B. mycoides, Salmonella Typhimurium, *Shigella flexneri, E. coli* DH5a, *E. coli* BL21 (DE3)	37℃ / 250 rpm / aerobic	Luria-Bertani (LB)
Staphylococcus aureus, Escherichia coli ,E. coli O157:H7, *Pseudomonas aeruginosa, P. putida, Yersinia enterocolitica, Cronobacter sakazakii*	37℃ / 250 rpm / aerobic	Bacto™ Tryptic Soy Broth Soybean-Casein Digest Medium
Geobacillus stearothermophilus, Weizmannia coagulans	50℃ / 250 rpm / aerobic	Bacto™ Tryptic Soy Broth Soybean-Casein Digest Medium
Levilactobacillus brevis	30℃ / 250 rpm / aerobic	Difco™ Lactobacilli de Man, Rogosa and Sharpe (MRS)
Clostridium perfringens	37℃ / anaerobic	Brain Heart Infusion (BHI)

Table S2. Plasmids and primers used in this study.

Plasmids or primers	Description or sequences (5′ to 3′)
**Plasmids**	
pET28a	pET28a Kan^r
pET28a::EGFP only	pET28a Kan^r; EGFP (NdeI / BamHI)
pET28a::PlyJBA6	pET28a Kan^r; PlyJBA6 (NcoI / SalI)
pET28a::EGFP::CBD	pET28a Kan^r; EGFP (NdeI / BamHI); PlyJBA6_CBD (BamHI / HindIII)
**Primers**	
fNco_PlyJBA6	gcg CCATGG gc ATGCAAATTTCACAAGCGGGCA
rSal_PlyJBA6	gcg GTCGAC ACTTAATCTAATTGTTTGACCTACTTTTATATG
fBamH_JBA6CBD	gcg GGATCC ACATCGTCTACTAAAACAACACCTAAGTATAAGGTG
rHind_JBA6CBD	gcg AAGCTT TCAACTTAATCTAATTGTTTGACCTACTTTTATATGATTT

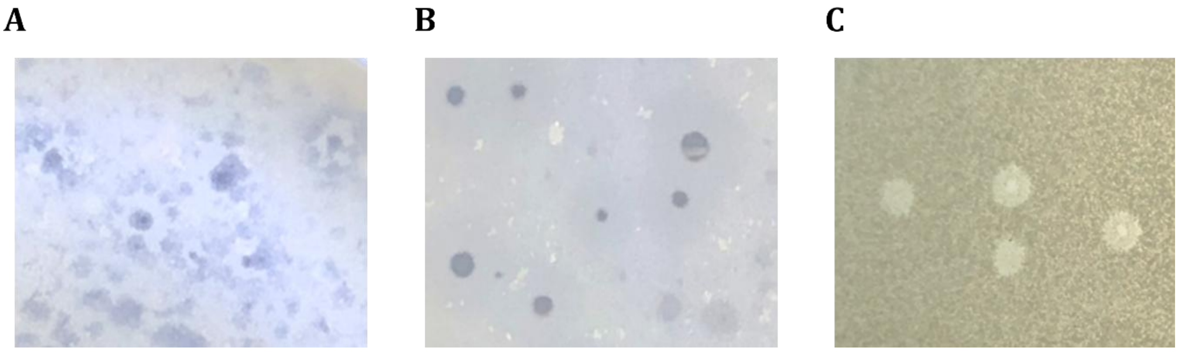

Fig. S1.

Plaque morphology of three *B. amyloliquefaciens* phages TBA3 (A), JBA3 (B), and JBA6 (C).

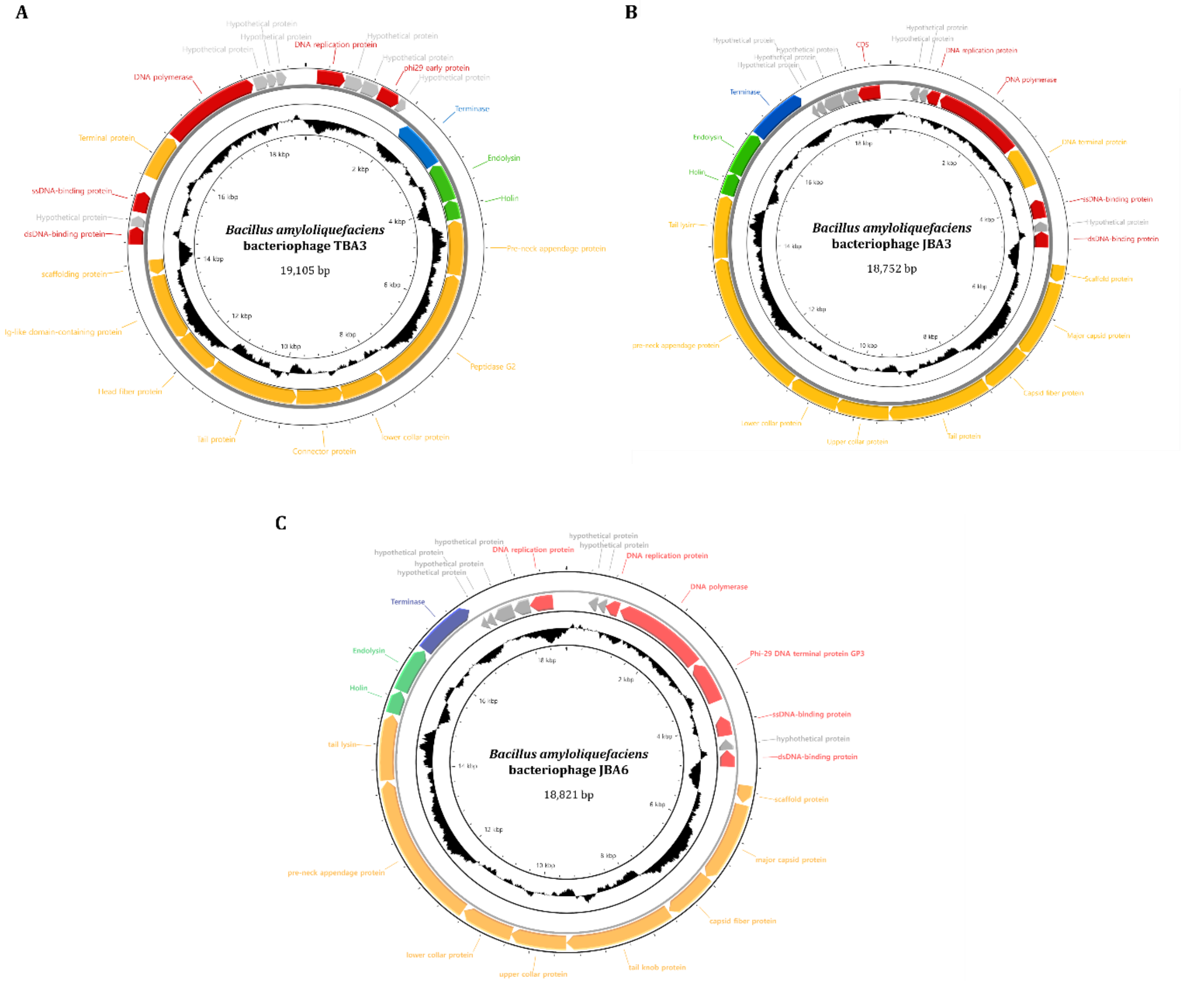

Fig. S2. The genome map of three *B. amyloliquefaciens* bacteriophages. Total 24 ORFs of TBA3 (A), JBA3 (B), and JBA6 (C) were predicted, respectively. The complete genome and predicted ORFs were visualized into circular maps with CGView. Each color of the arrow (ORF) indicates its function: Red, Nucleotide metabolism; Yellow, Structural protein; Green, Host lysis; Blue, Packaging: and Grey, Hypothetical protein. The inner circle with the black graph is G+C content.

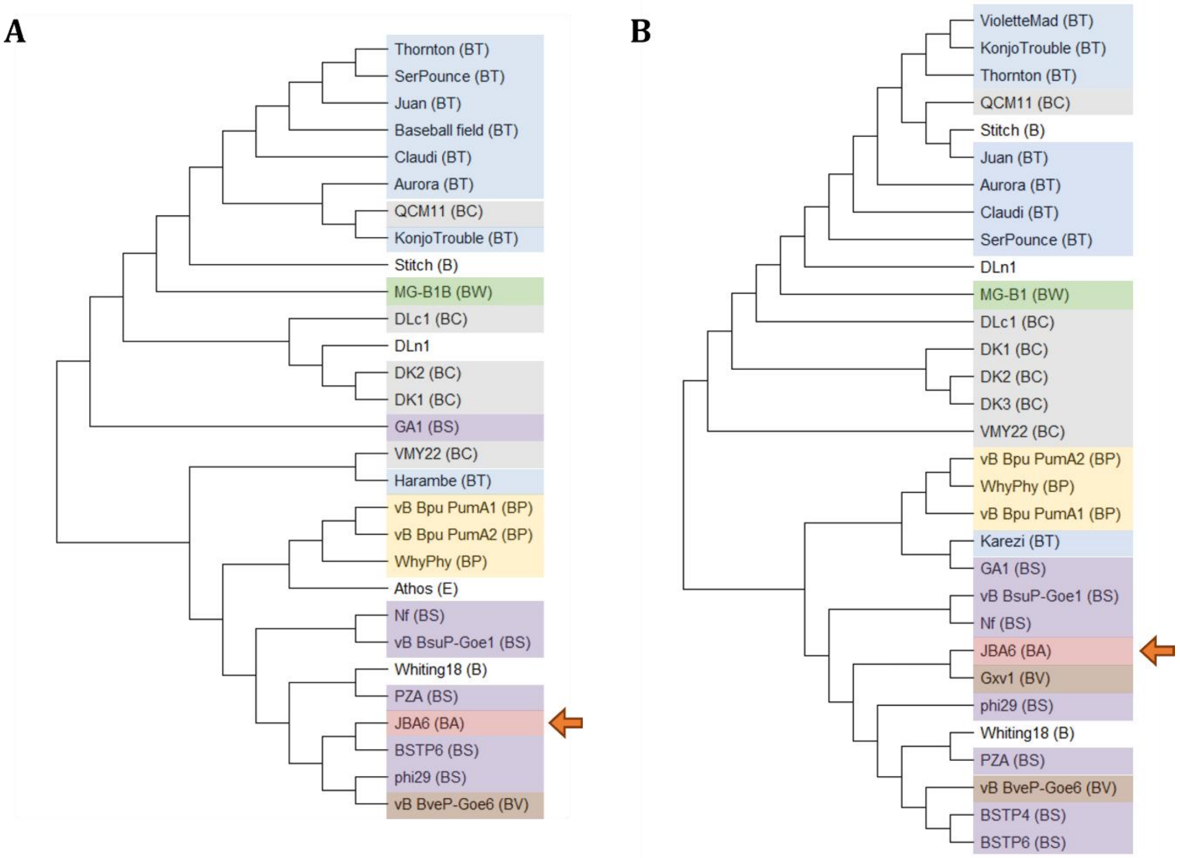

Fig. S3. Phylogenetic analysis of JBA6. The phylogenetic tree was constructed using the neighbor-joining analysis of the terminase large subunit (**A**) and the major capsid protein (**B**). The colored shadings over each phage name indicate the host species: B, *Bacillus* spp.; BT, *B. thuringiensis*; BC, *B. cereus*; BW, *B. weihenstephanensis*; BS, *B. subtilis*; BP, *B. pumilus*; BA, *B. amyloliquefaciens;* BV, *B. velezensis*; E, *Enterococcus*

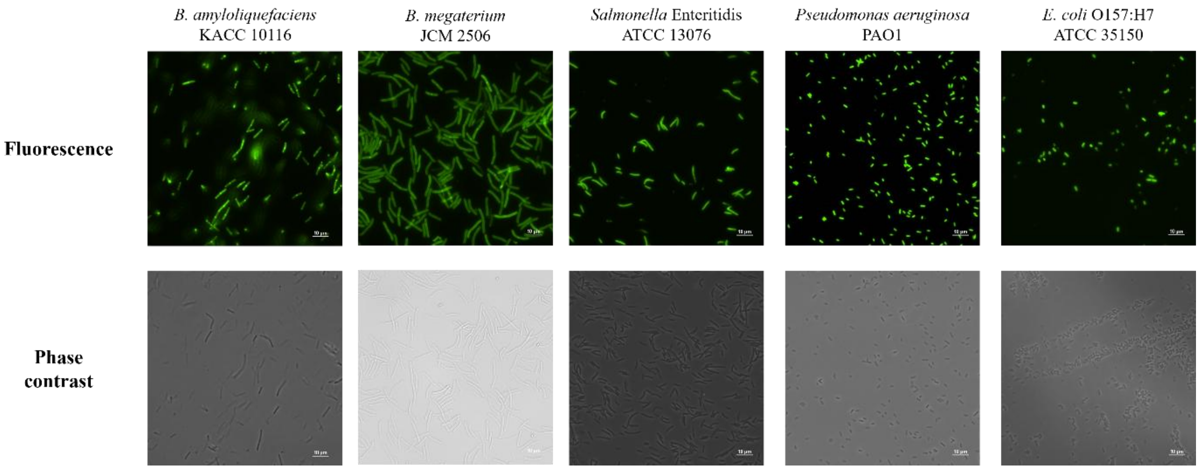

Fig. S4. Cell wall-binding analysis of EGFP::JBA6_CBD towards diverse bacterial cells. Cell wall binding activity of PlyJBA6_CBD was confirmed using fluorescence microscopy.
